# Exposure to fibrillar proteins leads to widespread infiltration but only mild tau pathology in cortical organoids

**DOI:** 10.1016/j.isci.2026.115819

**Published:** 2026-04-20

**Authors:** Abdulkhalek Dakhel, Tobias Mothes, Khalid Eltom, Wojciech P. Michno, Anna Erlandsson

**Affiliations:** 1 Department of Public Health and Caring Sciences, Molecular Geriatrics, Uppsala University, 75185 Uppsala, Sweden; 2 Science for Life Laboratory, Uppsala University, Uppsala, Sweden

**Keywords:** Biological sciences, Neuroscience, Stem cells research, Techniques in neuroscience

## Abstract

Alzheimer’s disease (AD) and Parkinson’s disease (PD) are the most common neurodegenerative disorders, both characterized by accumulation of aggregated proteins. In AD, the pathological deposits consist predominantly of amyloid-beta (Aβ) and tau, while alpha-synuclein (αSYN) forms inclusions in PD. However, cross-seeding often generates mixed pathologies. Emerging evidence suggests a role of astrocytes in disease spreading, but the underlying mechanisms remain unclear, partly due to limitations of mouse models in replicating early human disease. To address this, we developed a human cerebral organoid platform to study early sporadic AD/PD events. We introduced fibrillar aggregates of αSYN, Aβ, and tau directly into organoids or via astrocytes pre-exposed to the aggregates. All proteins successfully penetrated the organoids with distinct morphology and distribution patterns. Twelve weeks post-exposure, organoids exposed to Aβ or αSYN-containing astrocytes showed the highest insoluble tau levels, but none developed robust tau pathology, highlighting limitations in organoid modeling of tau pathology.

## Highlights

• Cerebral organoids can be used for modeling early-stage aggregate spreading
• Aggregates of αSYN, Aβ, and tau show distinct organoid infiltration patterns
• Aβ and αSYN-containing astrocytes induce mild accumulation of insoluble tau
• Lack of aggregation-prone tau isoforms limits tau seeding in organoids

## Graphical abstract

**Figure:**
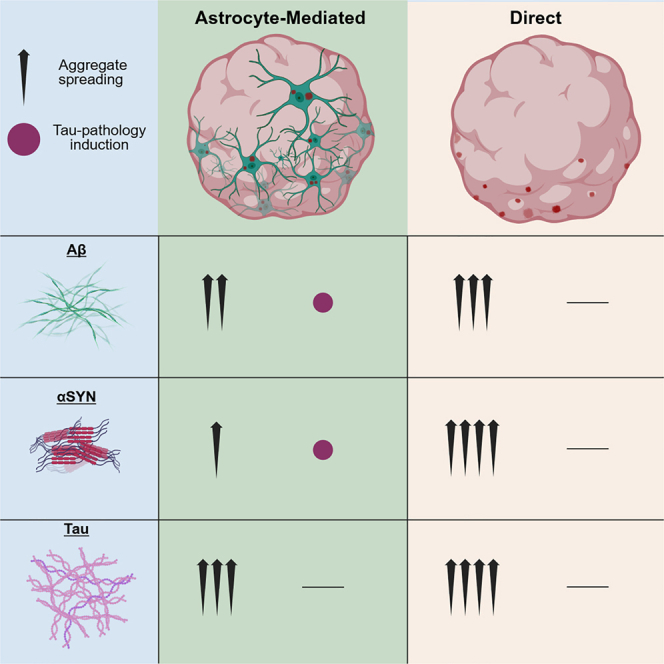

## Introduction

Alzheimer’s disease (AD) and Parkinson’s disease (PD) are common neurodegenerative disorders, marked by the accumulation of misfolded proteins in the brain. In AD, amyloid-beta (Aβ) and tau form the characteristic plaques and neurofibrillary tangles (NFTs), while alpha-synuclein (αSYN) forms Lewy bodies in PD. Tau also plays a central role in other tauopathies, including progressive supranuclear palsy and argyrophilic grain disease. The protein pathology is believed to propagate in a prion-like manner, beginning years before symptoms appear.^1^^,^^2^^,^^3^^,^^4^ Proposed spreading mechanisms include direct cell-to-cell transmission and extracellular vesicle (EV) secretion, but the exact cellular processes and the various cell types involved remain poorly understood. We have previously shown that astrocytes effectively engulf αSYN, Aβ, and tau aggregates, but fail to degrade them, leading to cellular stress and further propagation of pathogenic proteins.^5^^,^^6^^,^^7^

The complexity of the brain makes advanced research models for AD and PD challenging. Traditional mouse models have contributed significantly to our understanding of AD and PD. However, due to biological differences between human and mouse brain cells, these models often lack translatability.^8^^,^^9^^,^^10^ Recent breakthroughs in human induced pluripotent stem cell (hiPSC) technology have enabled the differentiation of human neurons and glial cells. In addition, the development of 3D cerebral organoids has provided platforms for more physiologically relevant systems.^11^ Compared to 2D cultures, 3D cell models show differences in cellular maturation, protein expression, and inflammatory responses.^12^^,^^13^^,^^14^^,^^15^ Moreover, organoid models have reported protein abnormalities associated with AD and PD, using patient-derived cells with early onset mutations^16^^,^^17^ or engineered cells overexpressing disease-related proteins.^18^^,^^19^ However, most AD/PD cases are sporadic and are not linked to specific mutations. Therefore, it is essential to develop translational models that better mimic the sporadic disease environment.

Here, we evaluated the use of cerebral organoids to investigate the spreading of non-mutated αSYN, Aβ, and tau aggregates. We examined the distribution patterns following direct exposure or co-incubation with astrocytes pre-exposed to aggregates. We also assessed whether these infiltrating fibrils could induce secondary tau pathology within the organoids.

## Results

### αSYN, Aβ, and tau aggregates infiltrate cerebral organoids and result in distinct morphological inclusions

**Figure 1:**
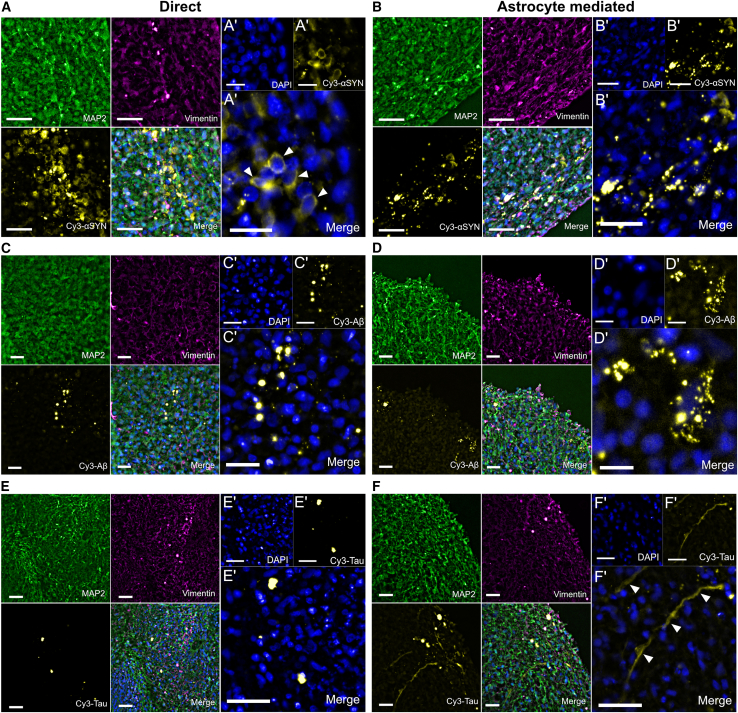
Cy3-labeled αSYN, Aβ, or tau aggregates successfully penetrate cortical organoids and exhibit morphologically distinct deposition patterns, depending on direct or astrocyte-mediated exposure Example images of fibril inclusions for each protein after four weeks of incubation. (A) Direct αSYN exposure. White arrows indicate internalized, intracellular Cy3-αSYN (A′). (B and B′) Astrocyte-mediated Cy3-αSYN exposure. (C and C′) Direct Cy3-Aβ exposure. (D and D′) Astrocyte-mediated Cy3-Aβ exposure. (E and E′) Direct Cy3-tau exposure. (F and F′) Astrocyte-mediated Cy3-tau exposure. White arrows indicate a tau positive projection infiltrating the organoid tissue. (A–F) scale bars, 25 μm. A′–F′ scale bars, 10 μm.

Building on previous studies that have shown successful induction of pathology in organoid systems using cells with strong genetic predispositions,^20^ we sought to develop a reproducible model that recapitulates sporadic AD/PD. We used a non-invasive approach, relying on exogenous fibril exposure to avoid mechanical damage to the organoids. At 70 days, cerebral organoids were exposed to Cy3-labeled fibrils of αSYN, Aβ, or tau (Figure S1A), either by direct addition to the media (direct exposure) or via pre-treated astrocytes with equivalent intracellular protein inclusions (astrocyte-mediated exposure) (Figures S1B and S1C). This ensured organoids remained intact, allowing us to follow the infiltration and spreading of pathogenic protein aggregates over time. Human astrocytes effectively engulf αSYN, Aβ, and tau aggregates, with minimal residual aggregates in the medium after 3 days.^5^^,^^6^^,^^7^ Hence, astrocytes were pre-treated for three days with the same amount of aggregates, used in direct exposure. Immunostainings confirmed the presence of expected cell types, positive for vimentin and the neuronal marker MAP2. A single dose of aggregates or pre-treated astrocytes was sufficient for Cy3-labeled protein aggregates to be detected inside organoids 4 weeks post-exposure (Figures 1A–1F). Following infiltration, clear morphological differences emerged between the αSYN, Aβ, and tau deposits, and between direct and astrocyte-mediated exposure. Direct αSYN exposure led to numerous small, intracellular aggregates (Figure 1A), with perinuclear deposition confirmed at higher magnification (Figure 1A′). This pattern was absent in astrocyte-mediated αSYN exposure (Figure 1B), which showed more punctate-like, clustered clumps (Figure 1B′). Direct Aβ exposure resulted in large inclusions diffused around and in-between cells throughout the tissue (Figures 1C and 1C′). Astrocyte-mediated Aβ showed similar morphology but limited distribution, suggesting that aggregates remained within astrocytes (Figures 1D and 1D′). Direct tau exposure resembled Aβ, with large, scattered inclusions (Figures 1E and 1E′). Interestingly, tau-astrocyte exposure also produced tau-containing projections extending from the infiltration sites toward the tissue core (Figures 1F and 1F′), likely originating from the added astrocytes, as they were absent in direct tau exposure.

### Organoid infiltration of Cy3-labeled αSYN, Aβ, or tau aggregates can be followed over time

Direct and astrocyte-mediated exposure to αSYN, Aβ or tau resulted in a Cy3 signal that could be visualized in a complete scan of the whole organoid section (Figures S2 and S3). Using a custom macro, we quantified the percentage of the Cy3-αSYN/Aβ/tau signal at incremental distances from the center (Figure S4). The Cy3 percentage was based on the total Cy3 signal measured for each individual organoid to allow comparisons between organoids with different levels of internalized aggregates. Additionally, to compare organoids of differing sizes, the penetration distance was relative to the largest organoid of the comparison. Each organoid was segmented into 5 regions from its outermost 20% (R1) part to the innermost 20% (R5). This allowed us to easily quantify the percentage of the Cy3-αSYN/Aβ/tau signal within each region.

### Free αSYN aggregates penetrate and disperse through cerebral organoids more efficiently than astrocyte-mediated αSYN

**Figure 2:**
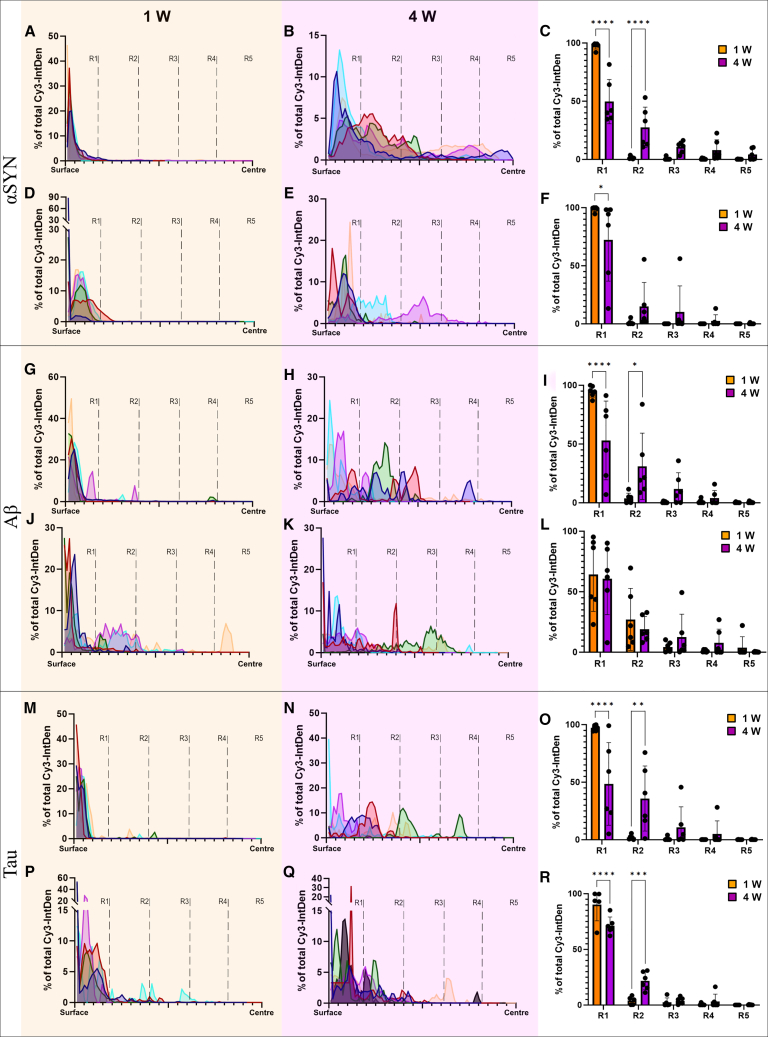
Distribution of Cy3-αSYN, Cy3-Aβ, and Cy3-tau in organoid tissue at 1 and 4 weeks following direct and astrocyte-mediated exposure Distribution of Cy3-signal after one (graphs highlighted in tan) and four (graphs highlighted in purple) weeks of incubation (see also Figures S2 and S3). Data are presented as a percentage of the total IntDen (determined for each organoid) at a certain location between the surface and the center of the organoid (illustrated in Figure S4). Individual organoids are represented by distinct colors. Representative examples of directly Cy3-αSYN, Cy3-Aβ, and Cy3-tau exposed organoids are shown in Figure S2 and representative examples of astrocyte-mediated exposed organoids are shown in Figure S3. The x*-*axis shows the proportional distance from the surface to the center of the largest organoid of the comparison. Dashed lines highlight each region (R1–5) from surface to center (R1 = outermost 20%, followed by R2, R3, R4, and R5 = innermost 20% as determined by the radius of each organoid). The y*-*axis shows the quantification of the IntDen within each region of the organoid. The graphs represent organoid exposed to (A) 1 week, direct αSYN, (B) 4 weeks, direct αSYN (C) and a statistical comparison of data presented in (A–B); (D) 1 week, astrocyte-mediated αSYN, (E) 4 weeks, astrocyte-mediated αSYN, (F) and a statistical comparison of data presented in (D–E); (G) 1 week, direct Aβ, (H) 4 weeks, direct Aβ, (I) and a statistical comparison of data presented in (G–H). (J); 1 week, astrocyte-mediated Aβ, (K) 4 weeks, astrocyte-mediated Aβ, (L) and a statistical comparison of data presented in (J–K); (M) 1 week, direct tau, (N) 4 weeks and a direct tau (O) and a statistical comparison of data presented in (M and N) (P) 1 week, astrocyte-mediated tau, (Q) 4 weeks, astrocyte-mediated tau (R) and a statistical comparison of data presented in (P and Q). Data in figures (C, F, I, L, O, and R) were from *n* = 6 organoids analyzed using two-way ANOVA, multiple comparison of mean values for each time point. Data are presented as mean ± SD. *p* values are presented as following; ∗*p* < 0.05, ∗∗*p* < 0.01, ∗∗∗*p* < 0.005, ∗∗∗∗*p* < 0.0001.

At one week, the αSYN aggregates were primarily found close to the surface of the organoid, penetrating only a few microns on average. However, 4 weeks after exposure, the Cy3-αSYN signal was highly distributed, and the aggregates had migrated more centrally throughout the entire organoid. Interestingly, the penetration was not uniform, but rather appeared to form continuous, centrally running channels (Figure S2A), suggesting a direct cell-to-cell transfer mode. This corresponds to our initial observation of αSYN inclusions being mainly intracellular (Figure 1A). Also, in the directly exposed organoids, the Cy3-αSYN signal distribution was very limited after one week of incubation, where the vast majority of αSYN was found at the crust of the outermost region R1 (Figure 2A). However, four weeks after exposure, the Cy3-αSYN signal had significantly migrated toward the center of the organoid. At this time point, the signal was detectable in all regions, indicating an efficient infiltration into the organoid tissue (Figure 2B). Statistical analysis of the Cy3 signal intensity confirmed the significant shift in distribution over time, where 50% of what resided in R1 at week 1 had relocated across R2-5 by week 4 (Figure 2C). Similar to direct exposure, astrocyte-mediated αSYN was almost exclusively detected in R1 after one week of incubation (Figure 2D). At four weeks, we observed some migration toward the inner regions, but the average penetration was low, compared to the pattern seen in direct αSYN exposed organoids (Figure 2E). Statistical analysis confirmed penetration toward the deeper layers, although most of the Cy3-αSYN signal remained in R1 (Figure 2F).

### Both direct and astrocyte-mediated Aβ exposure result in distinct penetration in cortical organoids

One week following direct exposure to Aβ, the aggregates were primarily located close to the surface of the organoid (Figure 2G). At this time, 96% of the protein aggregates were occupying the outer R1 region, indicating very limited infiltration. After 4 weeks, the distribution had significantly shifted toward the center of the organoid (Figure 2H). Similar to αSYN, around 50% of the Cy3-Aβ signal was found across R2 to R5 at the later time point, indicating a gradual infiltration of the free Aβ aggregates (Figure 2I). Quantification of Cy3-Aβ in the different regions confirmed a significant shift in distribution over time (Figure 2I). Interestingly, astrocyte-mediated Aβ showed penetration already after one week of incubation, with only 65% of the protein remaining within R1 (Figure 2J). However, the pattern was rather unchanged at 4 weeks (Figure 2K). Quantification of the regional distribution confirmed no significant changes between the time points, suggesting that most of the penetration took place already during the first week (Figure 2L).

### Astrocytes-mediated tau penetration is initially more effective, but free tau spreads more over time

In accordance with αSYN and Aβ, free tau aggregates were situated at the organoid surface, showing only minimal penetration after one week (Figure 2M). At this time, 96% of the signal remained in the outer half of R1. However, over time, tau became evenly distributed in the organoid tissue (Figure 2N), with about 45% remaining in R1 at four weeks (Figure 2O). Astrocyte-mediated tau spreading showed slightly more penetration at one week (Figure 2P), with 90% of the tau aggregates remaining within R1. However, even after four weeks, 72% of the astrocyte-mediated tau still remained in R1 (Figure 2Q). Quantification of the regional distribution verified this shift, with free tau spreading more effectively than astrocyte-mediated tau over time (Figure 2R).

### Immunostainings showed inconsistent pathological patterns in organoids following exposure to free and astrocyte-mediated αSYN, Aβ and tau fibrils

**Figure 3:**
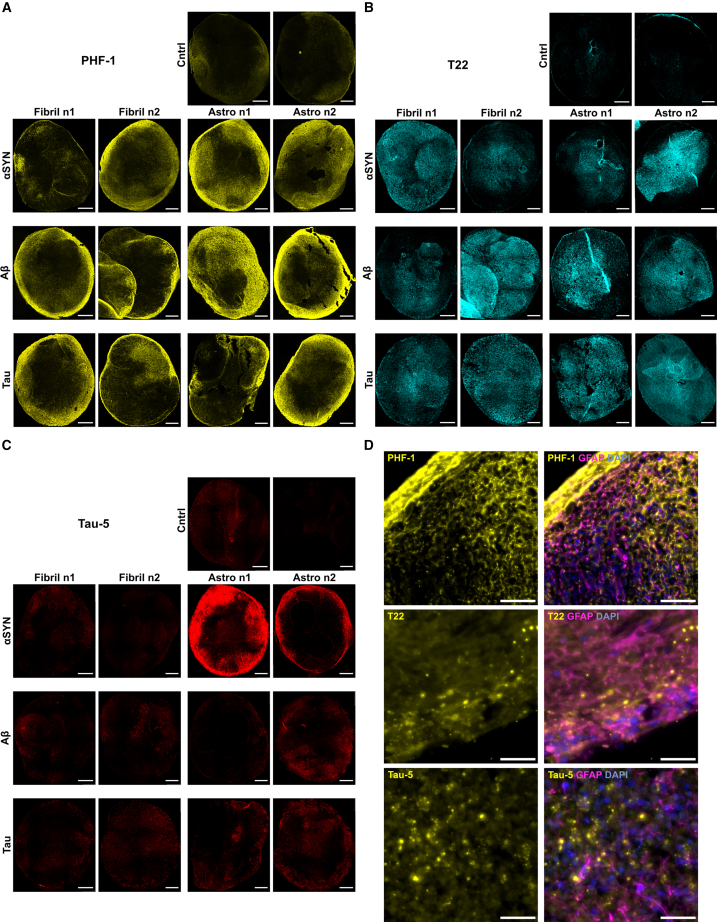
Tau staining patterns in organoids display heterogeneity between and within different treatment groups (A–C) Shows the expression pattern of PHF-1 (A), T22 (B), and tau-5 (C) across the different samples following αSYN, Aβ, and tau exposure; both direct (Fibril) and astrocyte-mediated (Astro). Pictures are from two individual organoids per group. (D) High magnification example images of the tau-positive signal patterns (PHF-1 is from astrocyte-mediated αSYN exposure, T22 and tau-5 are from direct Aβ-treated organoids). (A–C) scale bars, 250 μm, (D) scale bars, 25 μm.

Encouraged by the positive results from the infiltration assays, we investigated the capacity of the infiltrating aggregates to induce tau pathology in organoids. αSYN, Aβ, and tau fibrils have been suggested to seed/cross-seed endogenous tau aggregation in mouse models.^21^^,^^22^^,^^23^ We hypothesized that longer incubation would be needed for endogenous tau pathology inoculation. Hence, organoids were incubated for 12 weeks post-exposure (total age of 37 weeks). To assess tau pathology, antibodies detecting early tau oligomers (T22), late-stage phosphorylated tangles (PHF-1), and total tau (tau-5) were used. Surprisingly, all organoids, including the untreated controls, stained for PHF-1 (Figure 3A) and T22 (Figure 3B), markers of mature NFTs and tau pre-tangles, respectively. However, signal intensity/pattern varied across treatment groups, individual organoids, and different regions of the same organoid. Albeit positive, untreated organoids showed the weakest signal. Tau-5 staining was widely distributed, with prominent expression in organoids exposed to αSYN-containing astrocytes (Figure 3C). High-resolution imaging revealed diffuse cytoplasmic PHF-1 and intense perinuclear/extracellular T22, as well as tau-5-positive puncta in all conditions (Figure 3D).

### Western blot analysis revealed no robust increase in tau pathology in organoids following fibril exposure

**Figure 4:**
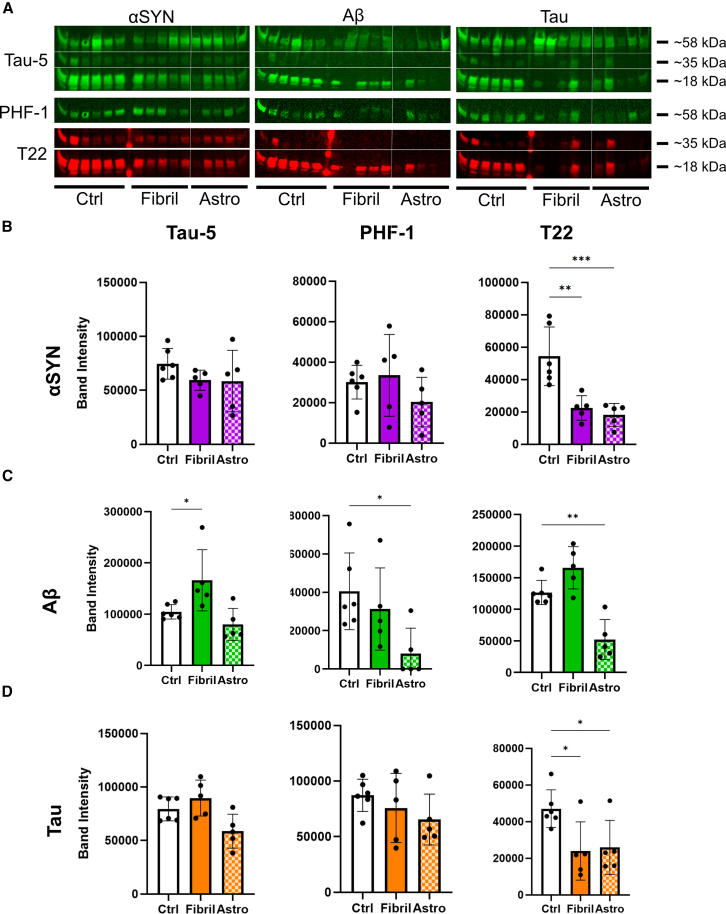
Pathology markers in the soluble organoid fraction after 12 weeks of exposure (A) Western blots of organoid lysate supernatant from αSYN, Aβ, and tau-exposed organoids (direct and astrocyte-mediated). Blots were stained for total tau (tau-5) and pathological phospho-tau (PHF-1, T22). Corresponding NOStain total protein normalization blots and uncut membranes are shown in Figure S5. (B) Quantification of αSYN blots. (C) Quantification of Aβ blots. (D) Quantification of tau blots. *n* = 6 individual organoids for control and *n* = 5 for the treated analyzed by one-way ANOVA, with multiple comparisons relative to control. Data are presented as mean ± SD, *p* values are presented as following: ∗*p* < 0.05, ∗∗*p* < 0.01, ∗∗∗*p* < 0.005.

**Figure 5:**
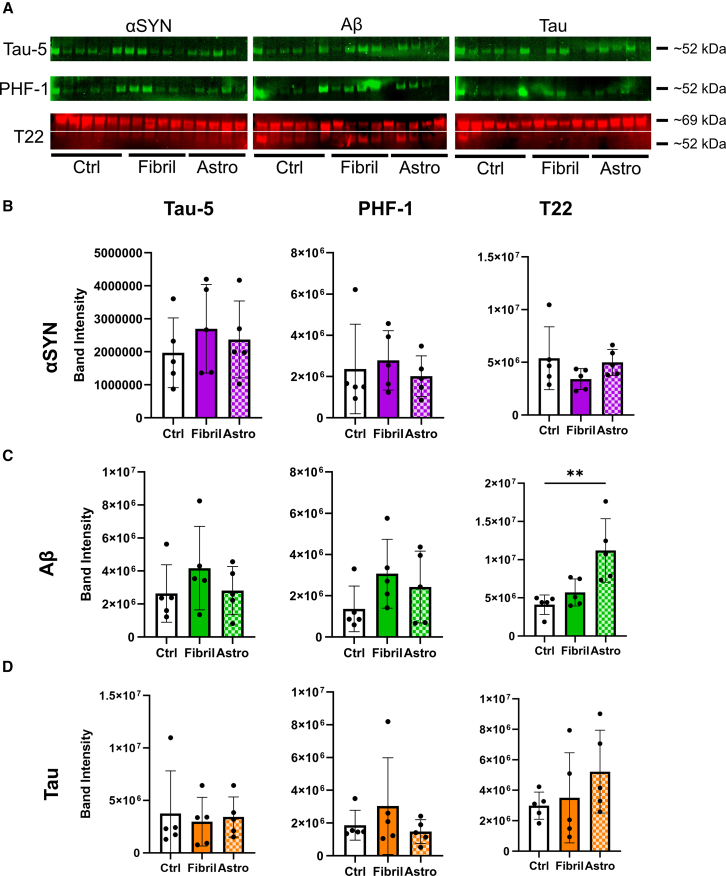
Pathology markers in the insoluble organoid fraction after 12 weeks of exposure (A) Western blots of organoid insoluble pellets from αSYN, Aβ and tau exposed organoids (direct and astrocyte-mediated). Blots are stained for total tau (tau-5) and pathological phospho-tau (PHF-1, T22). Corresponding NOStain total protein normalization blots and uncut membranes are shown in Figure S6. (B) Quantification of αSYN blots. (C) Quantification of Aβ blots. (D) Quantification of tau blots. *n* = 5 individual organoids analyzed by one-way ANOVA, with multiple comparisons relative to control. Data are presented as mean ± SD,∗∗*p* < 0.01.

Due to the heterogeneous IHC results, we used an alternative method to quantify total pathology per organoid across groups. Western blot analysis was conducted on the lysis buffer-soluble supernatant (hereafter referred to as the soluble fraction) (Figures 4 and S5) and the remaining insoluble pellet (the insoluble fraction) (Figures 5 and S6). In agreement with IHC data, blot quantifications revealed strong signals in both treated and control organoids (Figure 4A). Notably, the soluble fraction showed the highest values in unexposed organoids (Figures 4B–4D), while the insoluble fraction aligned more closely with IHC results (Figures 5A–5D). Overall, treatment responses were low, with a high sample-to-sample variation. However, organoids exposed to Aβ-containing astrocytes showed a significant increase in insoluble T22-positive tau deposits (Figure 5C), and comparison of the insoluble to soluble tau ratio revealed a significant increase in organoids exposed to αSYN- and Aβ-astrocytes (Figures S7A–S7C). Additional analysis of pathology using thioflavin T staining confirmed the presence of scattered amyloid aggregates in fibril-exposed organoids (Figure S8A). The thioflavin T-positive structures were most pronounced in organoids exposed to Aβ-astrocytes and to a lesser degree αSYN-astrocytes (Figure S8B), which corresponded to the western blot data. In conclusion, direct or indirect fibril exposure did not induce a consistent tau pathology profile in cortical organoids across the different treatments, despite signs of mild pathological changes caused by astrocyte-mediated delivery of pathological proteins.

### High background expression of pathological markers, apoptosis, and lack of aggregation prone tau isoforms limit the organoid model

**Figure 6:**
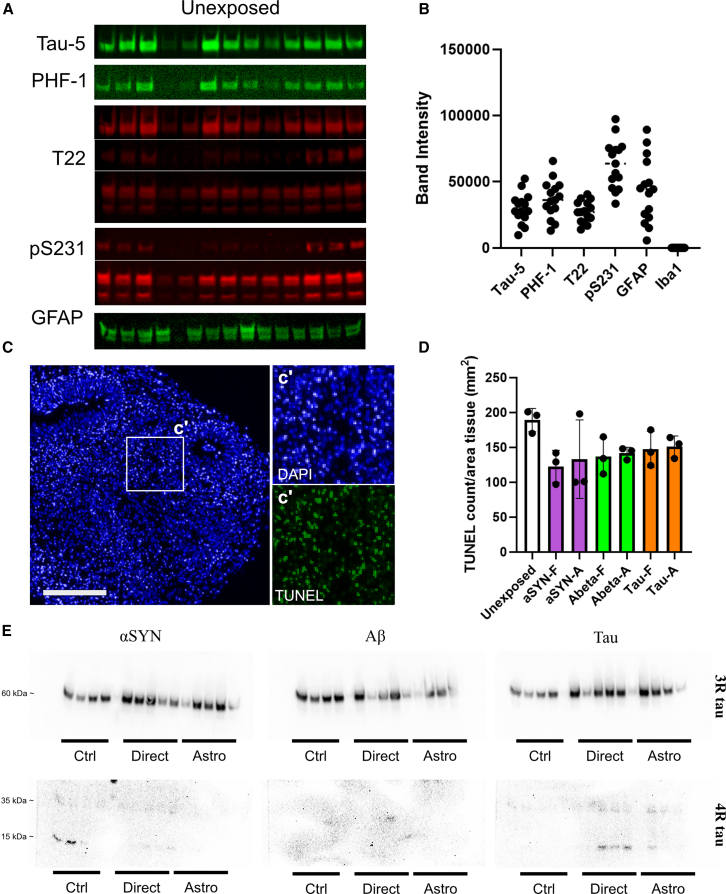
Control organoids display high levels of apoptosis and native tau pathology but lack full-length tau isoforms (A) Western blot analysis of unexposed organoids shows a positive signal for pathological tau (PHF-1, T22, pS231) and the astrocytic marker GFAP. Corresponding NOStain total protein normalization blots and uncut membranes are shown in Figure S9. (B) Relative band intensity demonstrates large variations between the native organoids. Data are presented as values for individual organoids (*n* = 15), and lines represent the median. (C) Representative images from apoptotic, TUNEL-labeled cells in organoids. (D) Quantification of TUNEL-positive cells in control and fibril-exposed organoids *n* = 3 individual organoids. Data are presented as mean ± SD. (E) Western blot analysis demonstrates that 3R-tau is the predominant tau isoform in 26 weeks organoids and that 4R-tau isoforms can barely be detected. Corresponding NOStain total protein normalization blots and uncut membranes are shown in Figure S9. Scale bars, 250 μm.

Next, we were prompted to investigate factors contributing to the absence of a robust pathological response. To verify high levels of pathological markers in controls, we analyzed 16 age-matched naive organoids (Figures 6A and S9). Although variations were detected, all of them showed significant pathological tau, including PHF-1, T22, pS231, and the astrocytic marker GFAP (Figure 6B). We previously showed that apoptosis of control neurons induces tau pathology in 2D cultures^5^ and hypothesized that tau pathology in unexposed organoids may result from apoptosis over time. To investigate this, we performed a TUNEL assay on sections from unexposed and fibril-treated organoids. Quantification of TUNEL-positive nuclei confirmed apoptosis in all tested organoids (Figures 6C and 6D). Interestingly, the highest counts were found in the unexposed organoids, although differences were small (Figure 6D). Hence, we suggest that high baseline pathology may stem from inherent cell death and degeneration in the organoid core during long-term culturing. Pathological features have previously been observed in organoids during prolonged growth.^20^ We then characterized the endogenous tau phenotype via western blot, using isoform-specific antibodies. Importantly, organoids showed almost no expression of full-length tau (2N4R). Nearly all detectable endogenous tau corresponded to three-repeat isoforms (2N3R, 1N3R, or 0N3R) (Figures 6E and S9), likely influencing the ability of exogenous tau to seed/co-aggregate with native tau. Thus, while the organoid model is efficient for studying early-stage amyloid protein spreading events, inherent cell death during extended culturing and limited tau isoform diversity limit its ability to model tau seeding and pathology.

## Discussion

Here, we have evaluated the use of genetically naive cerebral organoids in modeling important aspects of neurodegenerative disease pathology. We have investigated the model’s applicability by studying infiltration of unmutated pathological αSYN, Aβ, and tau protein aggregates, as well as their potential to mimic subsequent propagation of tau pathology. Organoids have been previously used to simulate vital junctions in the disease transmission process, where a viable propagation milieu is necessary. Liver organoids successfully replicated the full hepatitis B virus infection and replication cycle upon exogenous exposure to the recombinant virus.^24^ Moreover, brain organoid systems were used to study the lethal property of glioblastomas to invade the surrounding brain tissue.^25^ Prion-like seeding of disease-associated fibrillary proteins is a fundamental aspect of the neurodegeneration propagation.^26^ This study has used the organoid’s ability to take up and transfer protein aggregates to study this process, setting the stage for further in-depth investigation into the mechanisms governing aggregate progression.

The organoid system successfully recapitulated the spatial distribution patterns of protein aggregates in the sporadic AD and PD brain. αSYN localized mainly in perinuclear regions, and tau extended into cellular projections resembling astrocytic deposits.^27^ Aβ deposits, although typically extracellular, also showed evidence of cell-mediated transport. Importantly, our model highlights astrocytes as key players in disease progression. Tau deposits in astrocytes are frequently found in AD and other tauopathies.^28^^,^^29^ We have previously shown that astrocytes modified internalized aggregates and facilitated the spread of proteoforms with enhanced seeding potential.^5^^,^^7^^,^^30^ In this study, astrocytes containing Aβ or αSYN were also able to induce cross-seeding of tau pathology in organoids.

While IHC revealed increased phosphorylated tau in the fibril-treated organoids, immunoblotting did not confirm this result, accentuating limitations of imaging-dependent assessment of neuropathology.^31^ The outcome of such analysis is often limited to the presence/absence of the investigated epitope, without information about the protein size or folding. Western blot analysis, however, provided a clearer tau profile, distinguishing soluble from insoluble fractions—an important marker of early-stage pathology, as brain aging is associated with a shift toward insolubility.^32^ This change is subtle and precedes the development of a full-blown aggregation pathology, but can be an important indicator of immature pathology, especially in early-stage disease models.

Importantly, aggregate-exposed organoids, but also naive control organoids, expressed high basal levels of the pathology markers assessed, rendering it difficult to establish a direct causal relationship. Similar findings were reported by Wendt et al.,^33^ where chronic Aβ exposure failed to induce clear tau pathology. Moreover, reliable detection of pathological tau is highly dependent on the choice of antibodies used. Here, we investigated T22 and PHF-1 tau reactivity as proxies for early and late tau pathology in organoids. T22 was originally reported to react exclusively with pathological tau in its early oligomeric form, distinguishing it from tau monomers and late tangles.^34^ Our western blot data, however, showed extensive reactivity of T22 with monomeric tau species in both the soluble and insoluble organoid lysate fractions. These observations are in line with a recent study that challenged the proclaimed oligomer-specific reactivity of T22 and showed significant reactivity even in control brain extracts.^35^ Thus, results obtained using T22 in tau seeding studies in organoids should be interpreted with caution, as the pathology-independent signal could mask subtle pathological changes.

Notably, robust pathological tau accumulation in 3D models has only been observed in systems using cells with aggressive or early-onset mutations.^18^^,^^36^^,^^37^^,^^38^ Even in those cases, baseline tau phosphorylation was often present in controls, reaching comparable levels to disease conditions in some cases.^36^ While this baseline activity may reflect tau deposition seen in healthy individuals,^39^^,^^40^ it poses a challenge for modeling induced tau pathology. Furthermore, the cerebral organoids predominantly expressed the 3R-tau isoform, which is less aggregation-prone than 4R-tau.^41^ Since most human tauopathies involve 4R or mixed isoforms, this may limit the model’s responsiveness to exogenous fibrils and hinder downstream aggregation cascades. Taken together, our data show that aggregates of αSYN, Aβ, and tau can infiltrate cerebral organoids, but the spatial presence alone was not sufficient to elicit robust pathological profiles despite indications of early cross-seeding in astrocyte-mediated delivery. The high baseline pathology in the organoid system and its predominant 3R-tau expression restrict its applicability in studying seeding-mediated propagation of tau pathology.

### Limitations of the study

While organoids enabled assessment of fibril distribution dynamics, the pathological effects were generally mild, suggesting that the model was not optimally suited to capture downstream disease mechanisms under the tested conditions. The intrinsic degenerative progression in organoids may mask experimental effects, complicating the distinction between baseline changes and pathology-driven alterations within the 12-week time frame. A higher pathological burden or longer observation period may therefore be required to induce clear effects. Furthermore, this study relied on the fluorescent signal from pre-labeled fibrils to track infiltration and distribution, as this approach currently provides the most accurate and controlled means of tracking exogenously introduced aggregates. Alternative detection tools, such as commercially available antibodies, remain limited in their capacity to identify modified protein forms, especially following uptake by astrocytes. The development of more sensitive and comprehensive techniques will be important to assess propagation dynamics more conclusively in future studies.

## Resource availability

### Lead contact

Requests for further information and resources should be directed to and will be fulfilled by the lead contact, Anna Erlandsson (anna.erlandsson@uu.se).

### Materials availability

This study did not generate new unique reagents.

### Data and code availability

• Western blot images generated are included in the article and supplementary figures. Additional microscopy data reported here will be shared by the lead contact upon request.
• This study does not report original code.
• Any additional information required to reanalyze the data reported in this study is available from the lead contact upon request.

## Acknowledgments

We thank the iPS core facility at Karolinska Institutet for providing the Cntr9 NES-cells.

The TEM experiments were performed at Uppsala University BioVis platform and Figure S1B was created with BioRender.

This study was supported by grants from the 10.13039/501100004359Swedish Research Council (2021-02563, A.E.), the Swedish Alzheimer Foundation (AF-980656, A.E.), the Swedish Parkinson Foundation (1476/23), 10.13039/501100005701Åhlén Foundation (233044, A.E.), the 10.13039/501100003792Swedish Brain Foundation (FO2022-0083, A.E.), O and E Edla Johanssons Foundation (2021, A.E.), Olle Engkvist Byggmästare Foundation (215-0399, A.E.), the Dementia Foundation (2024, A.E.), Stohnes Foundation (2023), and the Swedish Fund for Research Without Animal Experiments (F2022-0004, A.E.), Konung Gustaf V:s och Drottning Victorias Stiftelse (2025).

## Author contributions

A.D. designed the study, optimized and performed experiments, interpreted the data, and wrote the manuscript; T.M. designed the study, optimized and performed experiments, interpreted the data, and wrote the manuscript; K.E. performed experiments and reviewed the manuscript; W.P.M. interpreted data, developed organoid culture protocols, and revised the manuscript; A.E. designed the study, interpreted data, coordinated the study, and wrote the manuscript. All authors have read and approved the final manuscript.

## Declaration of interests

The authors declare no competing interests.

## STAR★Methods

### Key resources table

**Table:** 

REAGENT or RESOURCE	SOURCE	IDENTIFIER
**Antibodies**		

Chicken polyclonal Anti-Vimentin	Merck	Cat#AB5733;RRID: AB_11212377
Rabbit polyclonal Anti-Tau (T22)	Merck	Cat#ABN454;RRID: AB_2888681
Mouse monoclonal Anti-Tau 3-repeat isoform RD3 (Clone 8E6/C11)	Merck	Cat#05–803;RRID: AB_310013
Mouse monoclonal Anti-Tau 4-repeat isoform RD4 (Clone 1E1/A6)	Merck	Cat#05–804;RRID: AB_310014
Chicken polyclonal Anti-GFAP	Abcam	Cat#ab4674;RRID: AB_304558
Rabbit monoclonal Anti-phosphoTau T231 (Clone EPR2488)	Abcam	Cat#ab151559;RRID:AB_2893278
Mouse monoclonal Anti-MAP2 (Clone 198A5)	Synaptic systems	Cat#188 011;RRID:AB_2147096
Mouse monoclonal Anti-Tau (TAU-5)	ThermoFisher	Cat#MA5-12808;RRID:AB_10980631
Mouse monoclonal Anti-Tau (PHF-1)	Gift from Dr. P. Davies through Prof. Martin Ingelsson,	Cat# PHF1;RRID:AB_2315150

**Chemicals, peptides, and recombinant proteins**		

Advanced DMEM/F12	ThermoFisher	11540446
L-Glutamine	ThermoFisher	25030-024
B27	ThermoFisher	11530536
non-essential amino acids	ThermoFisher	11140050
bFGF	ThermoFisher	11390832
CNTF	ThermoFisher	PHC7015
Trypsin-EDTA	ThermoFisher	15400054
Neurobasal medium	ThermoFisher	21103049
GlutaMAX	ThermoFisher	35050038
E8 medium	ThermoFisher	A1517001
E6 medium	ThermoFisher	A1516401
vitronectin	ThermoFisher	A14700
B27-noVitA	ThermoFisher	11500446
FGF2	ThermoFisher	10222253
EGF	ThermoFisher	17159651
NT-3	ThermoFisher	17109511
BDNF	ThermoFisher	17169321
Halt protease inhibitor cocktail	ThermoFisher	78430
Ever Brite Hardset Mounting medium	VWR	23004
Heregulin beta-1	Merck	SRP3055
IGF-1	Merck	SRP3069
Poly-L-Ornithine	Merck	P3655
Laminin	Merck	L2020
MES Hydrate	Merck	M2933
1,4-Dithiotheitol	Merck	D0632
Heparin	Merck	H3149
Thioflavin T	Merck	T3516
Activin A	Peprotech	120-14E
Y27	Cayman	Caym10005583
SB431542	Cayman	Caym13031
XAV939	Cayman	Caym13596
Dorsomorphine hydrochloride	Cayman	Caym21207
Recombinant human 441-tau	Anaspec	AS-55556
endotoxin-free monomeric α-synuclein	Anaspec	A5555
Flour 555-labeled Aβ monomers	Anaspec	AS-60480-01
Aβ monomers	Innovagen	SP-BA42-1

**Critical commercial assays**		

Pierce BCA protein kit	ThermoFisher	23225
Click-iT Plus TUNEL assay	ThermoFisher	C10619
No-Stain™ protein labeling reagent	ThermoFisher	A44717
Cy3-labeling kit	GE Healthcare	PA33000

**Experimental models: Cell lines**		

Human: Cntrl-9-II iPSC	Karolinska Institutet	RRID: CVCL_JL74

**Software and algorithms**		

FIJI (ImageJ)	Open Access	https://imagej.net/software/fiji/downloads
GraphPad Prism 10	GraphPad	https://www.graphpad.com/resources
Image Lab 6.1	Bio-Rad Laboratories Inc	https://www.bio-rad.com/en-se/product/image-lab-software?ID=KRE6P5E8Z
ImageStudie Ver 5.2	LI-COR Biosciences	https://www.licorbio.com/image-studio
Zen 3.5 (blue edition)	Zeiss	https://www.micro-shop.zeiss.com/en/de/softwarefinder/software-categories/zen-blue/
LAS X	Leica	https://www.leica-microsystems.com/products/microscope-software/p/leica-las-x-ls/

### Experimental model and study participant details

#### Cell lines

Human cortical organoids and astrocytes were derived from the male Ctrl-9-II human iPS cell line (RRID: CVCL_JL74) and tested for Identity, karyotype, and pluripotency, as well as mycoplasma at the iPS core facility at Karolinska Institutet.^42^ Cells were cultured in growth medium (as described below) under standard culturing conditions of 37°C, 5% CO_2_.

#### Culturing of human iPSC-derived cortical organoids

Human cortical organoids were generated from induced pluripotent stem cells (iPSC, CNTRL9 II cell line).^43^ The hiPS cells were cultured on 6-well cell-culture plates coated with 50 μg/mL vitronectin using E8+ medium (ThermoFisher). The medium was replaced every day and the cells were passaged at 70–80% confluency, using Accutase (ThermoFisher). The cells were resuspended in E8+ medium fortified with 1× Y27 (Cayman) and transferred as single cells to an AggreWell (Stemcell, 34411) (3M cells/well). The AggreWell plate was centrifuged at 300*g* for 5 min and placed in an incubator overnight to create spheroids. The spheroids were then gently collected and sieved through a cell strainer (Fisher Scientific, 10737821). The spheroids were kept on UltraLow adhesion 100 mm plates (ThermoFisher, 16855831) in E6+ medium (ThermoFisher) fortified with 10 μM SB431542 (Cayman), 0.25 μM XAV939 (Cayman) and 2.5 μM Dorsomorphine hydrochloride (Cayman). From the second day, the medium was changed every day. After 5 days, the medium was substituted for Neurobasal, containing 1% penicillin-streptomycin, 1× B27-noVitA and 1× GlutaMAX (ThermoFisher). For the first two weeks in Neurobasal, the medium was replaced every day, but for the coming four weeks it was replaced every other day. From day 6–24, 27 ng/mL FGF2 and 20 ng/mL EGF were added to the medium. From day 25–43, 20 ng/mL NT-3 and 20 ng/mL BDNF (ThermoFisher) were added to the medium. From day 44 onwards, the medium had no additional factors and it was changed twice a week.

#### Culturing of human iPSC-derived astrocytes

Human astrocytes were differentiated from neuroepithelial-like stem (NES) cells, produced from human induced pluripotent stem cells (iPSCs, Cntrl9 II cell line). To generate astrocytes, NES cells were cultured in Advanced DMEM/F12 supplemented with 1% penicillin-streptomycin, 1% L-glutamine, 1× B27 and 1× non-essential amino acids (ThermoFisher). The following factors were added to the medium right before use: 8 ng/mL bFGF (ThermoFisher), 10 ng/mL heregulin beta-1 (Merk), 10 ng/mL Activin A (Peprotech), 200 ng/mL IGF-1 (Merk). From week three of differentiation, 20 ng/mL of CNTF (ThermoFisher) was also included. A full medium change was performed every other day for the duration of the differentiation. Cells were cultured in cell culture flasks (Sarstedt) coated with 100 μg/mL poly-L-ornithine (Merk) and 50 μg/mL laminin (Merk), and seeded for experiments at 5,000 cells/cm^2^. Trypsin-EDTA 4% (Thermo Scientific, 10779413) was used for passaging the cells and the cells were differentiated for 28 days, prior to the start of experiments.

### Method details

#### Production of alpha-synuclein fibrils

Synthetic alpha-synuclein fibrils (αSYN-F) were generated using αSYN monomers (AnaSpec). Monomers were dissolved in PBS (5 mg/mL) and left on shake for 7 days at 37°C. The sample was centrifuged at 28,000*g*, 4°C for 30 min and the supernatant was removed. Pellet was resuspended to a stock concentration of 2 mg/mL and stored at −70°C until use. The fibrils were labeled using a Cy3-labelling kit (GE Healthcare) according to the manufacturer’s instructions. Prior to use, the fibrils were diluted 1:2 in PBS and sonicated for 1 min, 1 s ON/OFF, 20% AMP using a Sonics Vibra Cell sonicator.

#### Production of amyloid-beta fibrils

Synthetic amyloid-beta fibrils (Aβ-F) were generated using Cy3-conjugated Aβ monomers (AnaSpec) or unlabeled Aβ monomers (Innovagen). Monomers were dissolved in 10 nM NaOH/PBS to a concentration of 2 mg/mL and left to aggregate for 4 days on shake at 37°C. Pellet was resuspended in PBS to a stock concentration of 0.5 mg/mL and stored at −70°C until use. Prior to use, the fibrils were sonicated for 1 min, 1 s ON/OFF, 20% AMP using a Sonics Vibra Cell sonicator.

#### Production of tau fibrils

Synthetic tau fibrils (Tau-F) were generated using recombinant human 441-tau monomers (Anaspec). Monomers were initially dissolved (3 mg/mL) in 100 mM MES hydrate buffer, pH 6.5, containing 10 μM of 1,4-Dithiotheitol and 16.25 μM heparin (all from Merk) and incubated on slow shake at 37°C for 7 days. Tau aggregates were then centrifuged at 20,879×*g* for 30 min, 4°C and the pellets re-suspended in Phosphate-buffered saline, PBS (1 mg/mL). The fibrils were labeled using Amersham Cy3-labeling kit (GE Healthcare) according to the manufacturer’s instructions and stored at −70°C. Prior to the experiment, the fibrils were sonicated for 30 s, 1 s ON/OFF, 20% AMP using a Sonics Vibra Cell sonicator.

#### Transmission electron microscopy of synthetic protein fibrils

Characterisation of the produced αSYN-F, Aβ-F and Tau-F was achieved through negative staining and transmission electron microscopy (TEM) imaging. At the end of the fibrilization process, protein samples were spun at 16,000*g*–20,000*g* to isolate the formed insoluble fibrils. Each fibril pellet was resuspended to its corresponding stock concentration (mentioned above) and 5 μL sample was taken for TEM analysis. Samples were added on a glow discharged Formvar- and carbon-coated 200-mesh copper grid (Ted Pella). After 10 s, the excess solution was removed by blotting with a filter paper, and the grid was washed with drops of MilliQ water (2×). Samples were contrasted on a drop of 2% Uranyl acetate for 10 s, and the excess solution was removed by blotting with a filter paper, and the grid was left to air dry. Grids were imaged using either an HT7800, Hitachi, Japan EM operated at 100 kV with XAROSA camera (EMSIS GmbH, Germany) or a FEI Tecnaii G2 operated at 80 kV with an ORIUS SC200 CCD camera.

#### Exposure of organoids to protein aggregates and astrocytes with protein inclusions

Three days before exposure of organoids, astrocytes were treated with 500 nM αSYN-F, 200 nM Aβ-F or 200 nM Tau-F (Cy3-labelled or unlabelled). 10 weeks (infiltration assays - Cy3 labeled) Or 6 months (pathology induction assays - Cy3 unlabelled) old organoids were transferred to individual wells of a 96-well UltraLow adhesion plate (Fisher Scientific, 174932) containing 100 μL of medium. Then, another 100 μL of medium was added, including either 10 000 astrocytes (containing protein aggregates) or an equivalent amount of αSYN-F, Aβ-F or Tau-F. This was calculated by assuming 100% uptake after the three days of incubation, which was then used to estimate the total protein load per astrocyte. The organoids were cultured separately and the medium was replaced every other day. Organoids exposed to Cy3-labelled fibrils were fixed overnight in 4% PFA after 1 or 4 weeks of incubation (11 or 14 weeks in total). The next morning, they were transferred to a 30% sucrose solution until sectioning. Organoids exposed to unlabelled fibrils were kept for 12 weeks before lysation and fixation (37 weeks in total).

#### Immunohistochemistry (IHC)

Organoids were snap-frozen on dry ice and sectioned as 12 μm thick slices using a Leica CM1860 UV cryostat and placed on coverslips. The coverslips were set to dry overnight at RT and subsequently frozen until staining. Blocking and permeabilization were performed by incubation in 5% normal goat serum (NGS) and 0.1% Triton X-100 in PBS for 30 min at RT. Primary antibodies; Chicken polyclonal Anti-Vimentin, Rabbit polyclonal Anti-Tau (T22) (Merk), Mouse monoclonal Anti-MAP2 (Synaptic systems), Mouse monoclonal Anti-Tau (TAU-5) (ThermoFisher), Chicken polyclonal Anti-GFAP (Abcam) and Mouse monoclonal Anti-Tau (PHF-1) were diluted in 0.5% NGS 0.1% Triton X-100 in PBS and incubated overnight at 4°C. The sections were washed 3 × 10 min with PBS before incubation with secondary antibodies; AlexaFluor goat-anti mouse, rabbit or chicken; 488, 555 or 647 (1:200, Molecular Probes) diluted in 0.5% NGS and 0.1% Triton X-100 in PBS for 1 h at 37°C. For evaluation of apoptosis, the Click-iT Plus TUNEL assay (Invitrogen) was used according to the manufacturer’s recommendations. The sections were washed 3 × 5 min with 1× PBS and mounted using Ever Brite Hardset Mounting medium with DAPI (VWR). Images were captured using the Leica DMi8 microscope.

#### Western blot (WB)

For lysis, organoids were moved to individual wells containing ice-cold lysis buffer (20 mM Tris pH 7.5, 0.5% Triton X-100, 0.5% Deoxycholic acid, 150 mM NaCl, 10 mM EDTA, 30 mM Na4O7P2, supplemented with 1× Halt Protease Inhibitor Cocktail (ThermoFisher) where they got macerated into a homogenate using a cell scraper (Thermo Fisher, 99002). Organoid homogenates were transferred to LO-bind tubes and vigorously pipetted to dissociate remaining clumps and then incubated on ice for 60 min before being centrifuged at 12,000*g* for 30 min (4°C). The fraction solubilised by the lysis buffer was collected from the supernatant to be used for the soluble protein analyses. The lysis buffer-insoluble pellet was resuspended in an equal volume of 1%SDS and sonicated for 1 min, 2 s ON/1 s off at 25% AMP and used for analysis of the insoluble protein analyses. Total protein concentrations of cell lysates were determined using Pierce BCA protein assay kit (ThermoFisher) according to the manufacturer’s instructions. Protein samples were denatured by incubating with Bolt sample reducing agent (Invitrogen, B00009) in LDS sample buffer (Invitrogen, NP0007) for 5 min at 95°C. The samples were then loaded on a 4–12% Bis-Tris Plus Gel (Invitrogen, NW04125BOX) with 5 μL PageRuler Plus (ThermoFisher, 26619) protein ladder and run for 20 min at 200 V in MES SDS running buffer (ThermoFisher, B0002). Transfer onto mini PVDF transfer stacks (ThermoFisher, PB5240) was performed using the Invitrogen Power Blotter (PB0010) with mixed range setting. Blocking was performed with 5% bovine serum albumin (BSA) in Tris Buffered Saline-Tween20 (TBS-T) for 1 h at room temperature (RT). The membrane was then incubated with primary antibodies; Rabbit polyclonal Anti-Tau (T22), Mouse monoclonal Anti-Tau 3R, Mouse monoclonal Anti-Tau 4R (Merk), Chicken polyclonal Anti-GFAP, Rabbit monoclonal Anti-phosphoTau T231 (Abcam), Mouse monoclonal Anti-Tau (TAU-5) (ThermoFisher) or Mouse monoclonal Anti-Tau (PHF-1) diluted in 5% BSA TBS-T, at 4°C overnight, washed and incubated with secondary antibodies (goat anti-rabbit and anti-mouse DyLight 680, and goat anti-rabbit and anti-mouse DyLight 800, diluted 1:20000 in 5% BSA, TBS-T) for 1 h at RT. The signal was analyzed using an SA Odyssey (LI-COR). Band intensity was measured using the ImageStudio (LI-COR) or ImageLab (BIO-RAD) software. Each band intensity was normalised to the total protein in the corresponding lane using the no-stain Protein Labeling Reagent (ThermoFisher) imaged on a BIO-RAD ChemiDoc XRS+.

#### Thioflavin T labeling

A 10 mM stock solution of Thioflavin T (Merk) was prepared fresh right before staining and passed through a 0.2 μm filter. For amyloid aggregate labeling in organoids, the stock solution was diluted to a concentration of 20 μM in PBS and sectioned organoids were incubated with the solution on shake at RT for 20 min. Then, organoids were washed 2 × 5 min in PBS and mounted with Ever Brite Hardset Mounting medium with DAPI. Images were captured using the Leica DMi8 microscope.

### Quantification and statistical analysis

#### Image analysis

##### Spreading of αSYN, Aβ and tau aggregates

A custom macro for ImageJ was made to quantify the distribution of αSYN, Aβ and tau throughout the organoid. In short, a cellular marker (vimentin) was used to establish a distance map to identify the center and border of the organoid. The middle section of each organoid was used for the calculation. A string command was used to generate regions of interest (ROIs) from the border all the way into the center (each ROI had a present size of 25 pixels), using the distance map as a template. Each ROI was superimposed over the corresponding Cy3 image (fluorescently labeled αSYN/Aβ/Tau) to measure IntDen within each ROI. The IntDen within each ROI was normalized to the total IntDen for the entire organoid to yield a percentage within each ROI. Six organoids per condition and time point were analyzed.

The distribution data (“b-c, f-g” in Figures 2, 3, and 4) was plotted as a percentage of the total IntDen in each organoid, and a relative distance to the center of the largest organoid for that comparison. For statistical analysis, the data were also compiled into regions (R1 = outermost 20%, followed by R2, R3, R4 and R5 = innermost 20% of the surface-to-centre distance).

##### TUNEL assay

For the quantification of TUNEL assay tile images of the full organoid section (*n* = 3 per condition) were captured with a 40× objective. A custom ImageJ macro was used to quantify the TUNEL positive cells in relation to total cell nuclei (DAPI).

#### Western blot quantification

The intensity of the detected immunoreactive bands was measured using ImageStudio (LI-COR) or ImageLab (BIO-RAD) software. Each band was normalized to the total protein of that respective lane, using the No-Stain protein labeling reagent (ThermoFisher). After the proteins were transferred to the PVDF membrane, the membrane was washed in ultrapure water and incubated for 10 min in No-Stain protein labeling solution. Images were captured using the Odyssey SA (LI-COR) or ChemiDoc XRS+ (BIO-RAD).

#### Statistical analysis

All statistical analyses were performed in Graphpad Prism (v.10). The datasets were initially analyzed using the D’Agostino-Pearson omnibus and the Shapiro-Wilk normality tests prior to further analysis. The region distribution was then further analyzed with a two-way ANOVA with multiple comparisons of mean values between time points and Šidák multiple analysis correction. Western blots were analyzed using one-way ANOVA with multiple comparisons relative to the control band. A total of 5 organoids from each treatment group were used for the comparisons and an additional 16 unexposed organoids. *p*-values are presented as following; ∗*p* < 0.05, ∗∗*p* < 0.01, ∗∗∗*p* < 0.005, ∗∗∗∗*p* < 0.0001. Information about statistical tests for individual experiments are included in the figure legends.
