# Supplementary material for: Exposure to fibrillar proteins leads to widespread infiltration but only mild tau pathology in cortical organoids
Source: iScience. 2026 Apr 20;29(5):115819. doi: 10.1016/j.isci.2026.115819 (PMC13157081; doi:10.1016/j.isci.2026.115819)
Supplement: Document S1. Figures S1–S9 [file mmc1.pdf]

iScience, Volume *29*

Supplemental information

Exposure to fibrillar proteins leads to widespread infiltration but only mild tau pathology in cortical organoids

Abdulkhalek Dakhel, Tobias Mothes, Khalid Eltom, Wojciech P. Michno, and Anna Erlandsson

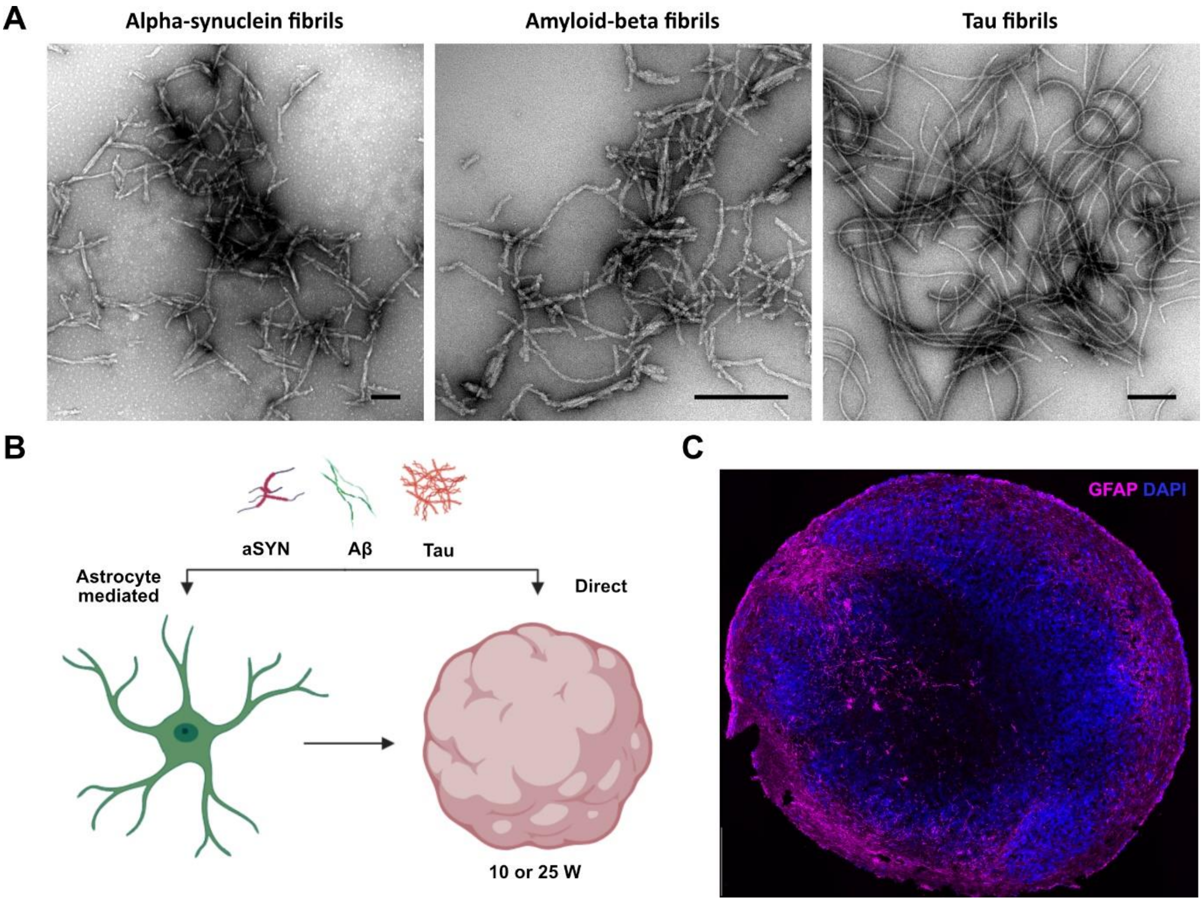

Figure S1. Schematic of experimental layout. Related to Figure 1. (**A**) Representative electron microscope images of synthetic αSYN, Aβ, and Tau fibrils. Scale bars: 200 nm. (**B**) 10 weeks old (infiltration assay) or 25 weeks old (pathology induction) human cortical organoids were either directly exposed to sonicated Cy3-labelled/unlabelled αSYN, Aβ, or tau fibrils, or to astrocytes pre-treated with the same proteins for three days. The organoids were further cultured for one or four weeks for organoids exposed to Cy3-labelled fibrils and 12 weeks for those exposed to unlabelled fibrils. (**C**) Representative ICC staining of cortical organoid showing the distribution of the astrocytic marker GFAP in 25 + 12 weeks organoids.

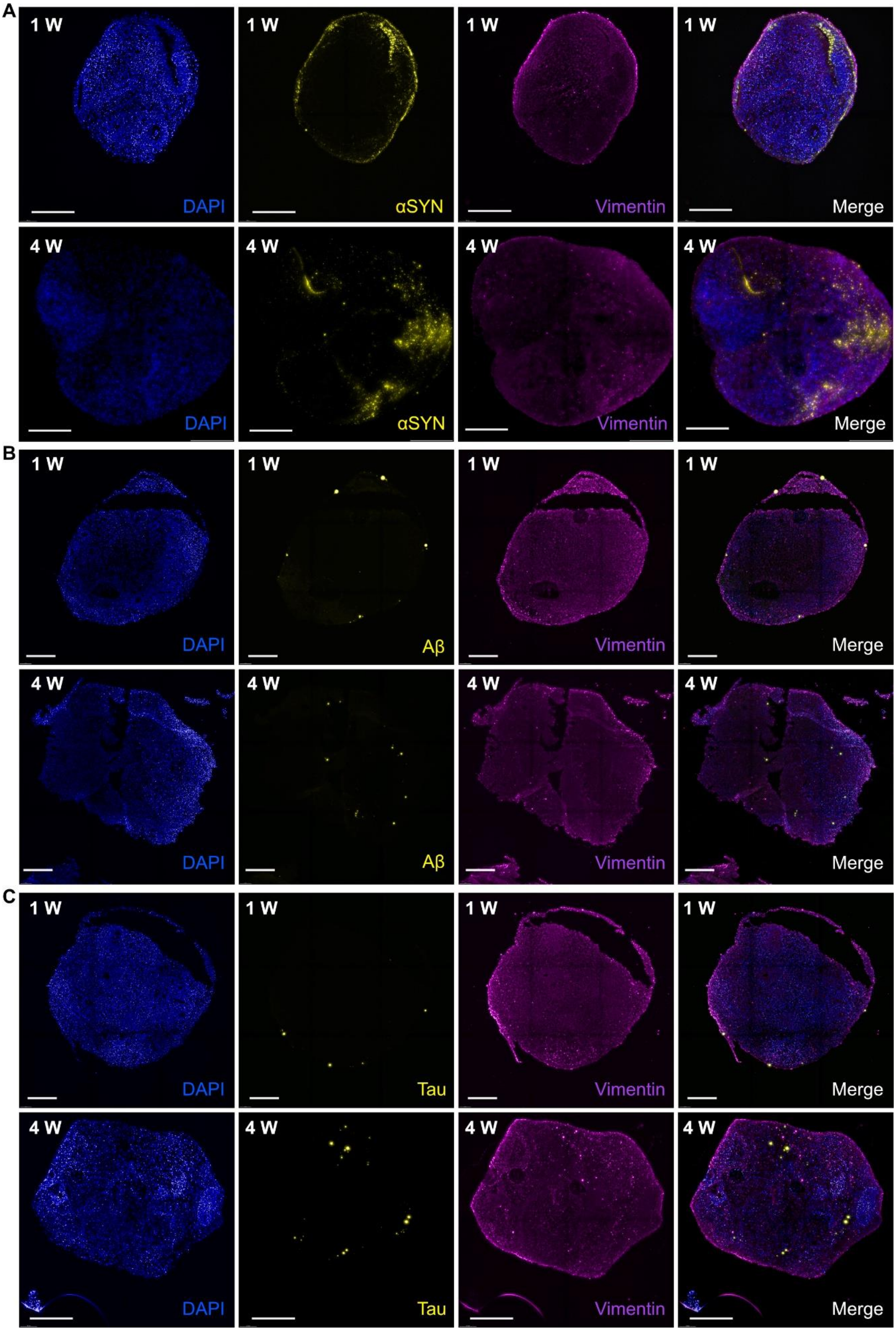

Figure S2. Example images of Cy3-signal distribution in organoids following direct fibril exposure. Related to Figure 2. Organoids exposed to (**A**) Cy3-αSYN, (**B**) Cy3-Aβ or (**C**) Cy3-Tau and fixed after one and four weeks of exposure. Data visualised in **Figure 2**. Scale bars = 250 µm.

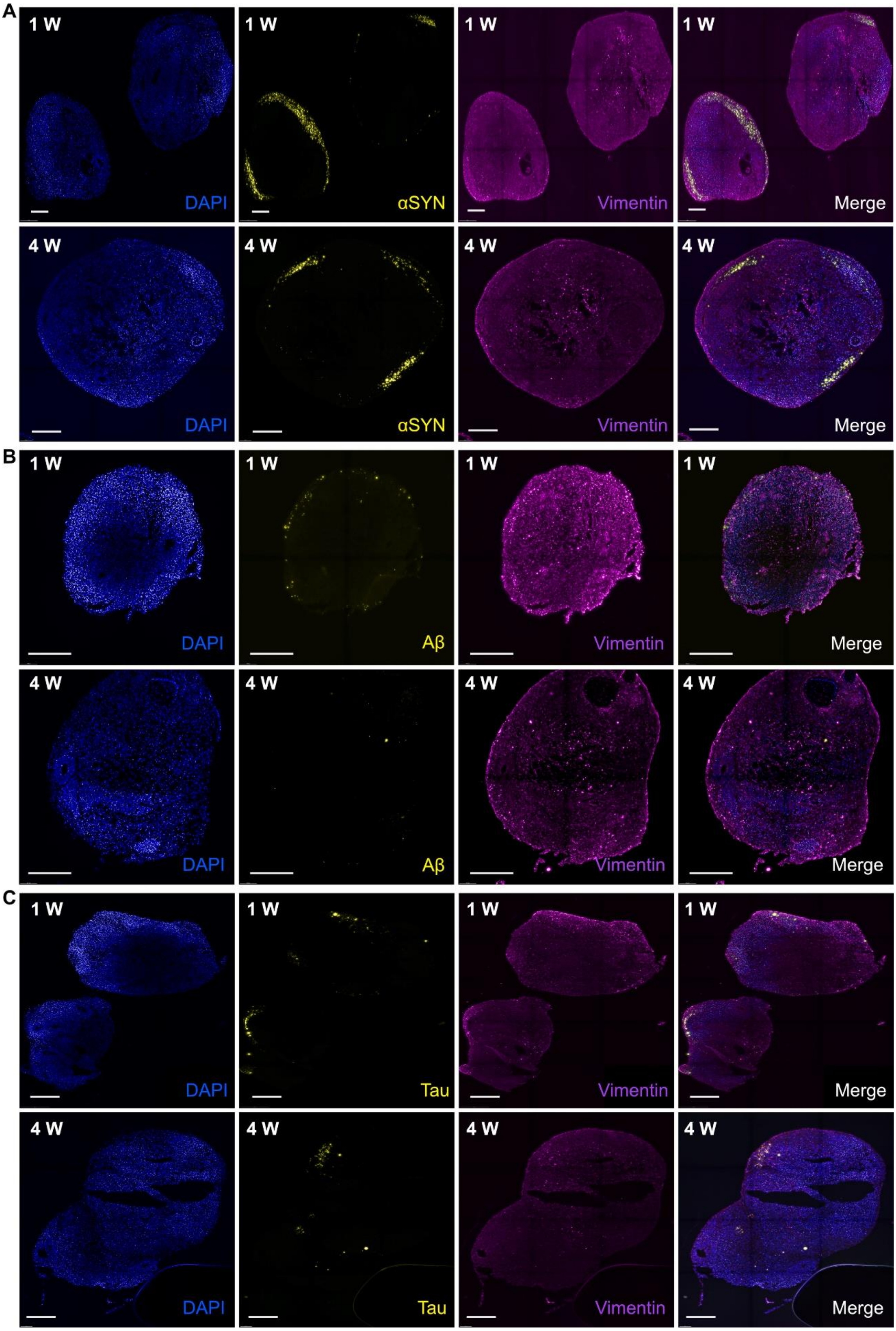

Figure S3. Example images of Cy3-signal distribution in organoids following astrocyte-mediated fibril exposure. Related to Figure 2. Organoids exposed to astrocytes containing (**A**) Cy3-αSYN, (**B**) Cy3-Aβ or (**C**) Cy3-Tau and fixed after one and four weeks of exposure. Data visualised in **Figure 2**. Scale bars = 250 µm.

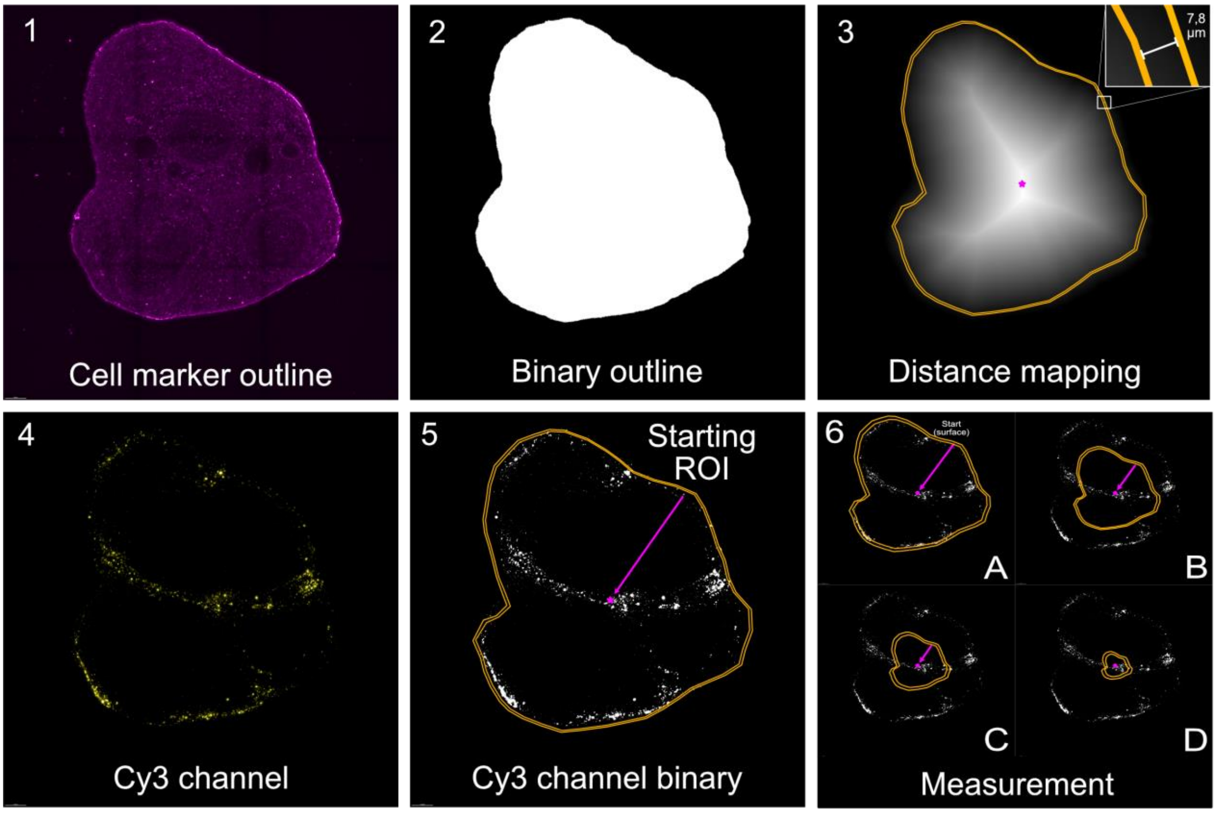

Figure S4. Visual explanation of the major steps of the custom macro used in this study. Related to Figure 2. Cell marker channel used for outlining the organoid (**1**). Outline converted to binary (**2**). Distance mapping of the outline to define the surface and the centre of the organoid. Purple star indicates the centre. The starting ROI (yellow region) was created using the distance map, set to a distance of 7.8 µm (**3**). Cy3 channel (representing αSYN, Aβ, and tau aggregates) (**4**). The Cy3 channel was converted into binary. Starting ROI was superimposed, the purple arrow indicates the direction and distance between the surface and the centre (**5**). Cy3 IntDen was measured within the superimposed ROI. A string function was used to sequentially move the ROI closer towards the centre, as indicated by A-D. This process contained as many sequences as required to reach the centre (**6**).

Figure 4A

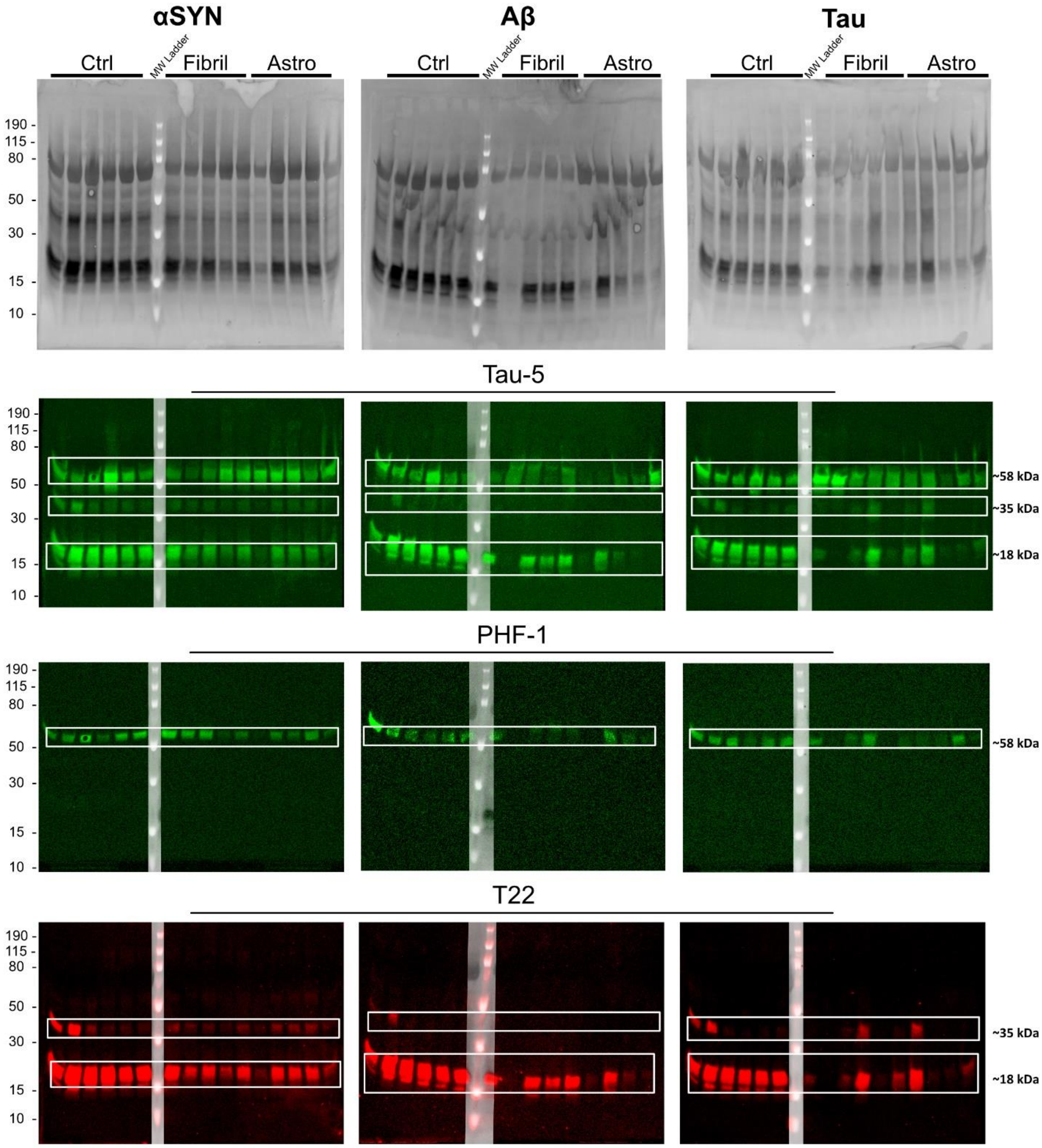

Figure S5. Related to Figure 4. Uncropped membranes and Nostain total protein loading controls of the membranes in **Figure 4a.** The loading control (top panel) image was superimposed on each corresponding signal image (lower panels) to show the bands' location in reference to the MW ladder.

Figure 5A

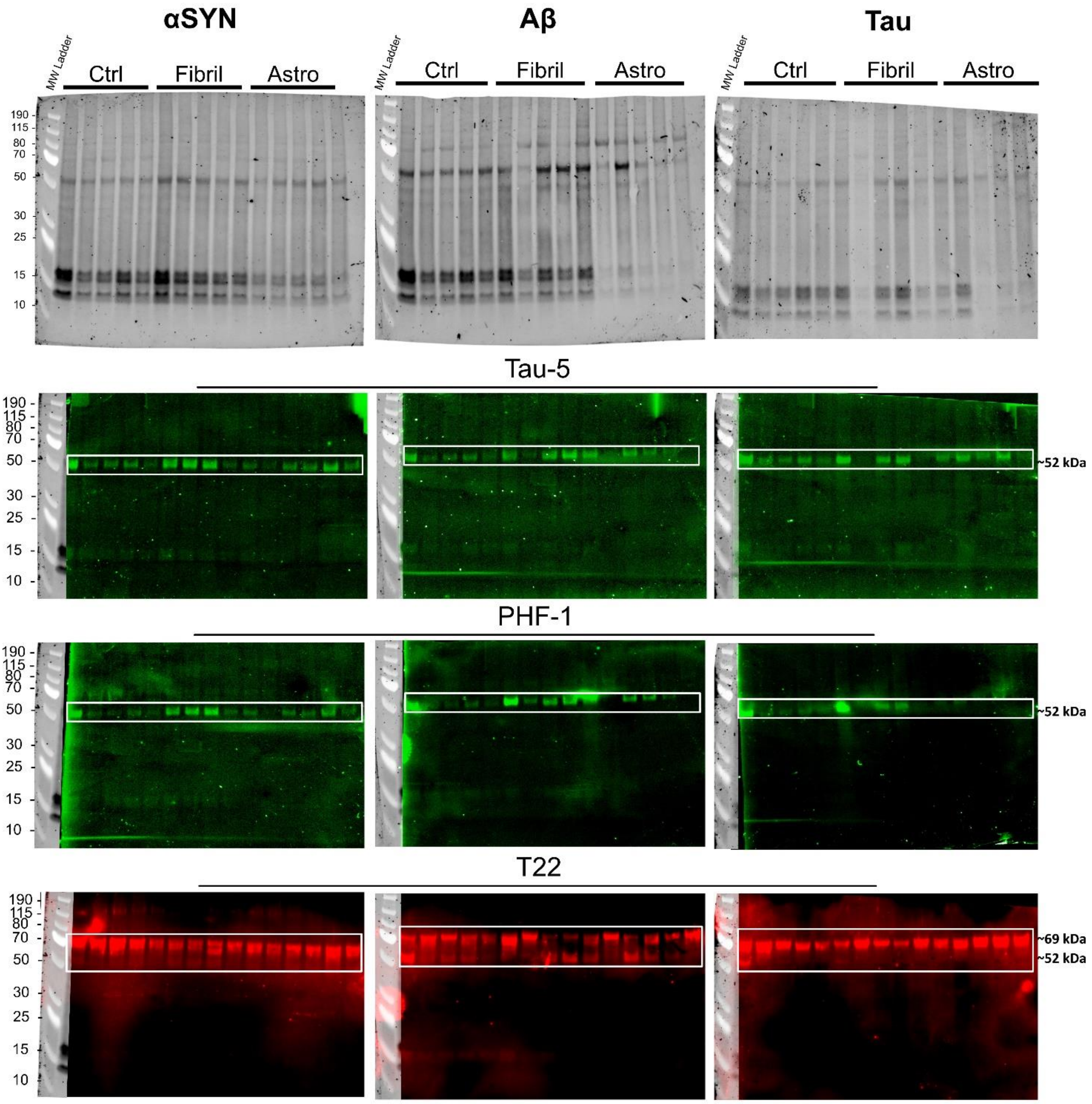

Figure S6. Related to Figure 5. Uncropped membranes and Nostain total protein loading controls of the membranes in **Figure 5a.** The loading control (top panel) image was superimposed on each corresponding signal image (lower panels) to show the bands' location in reference to the MW ladder.

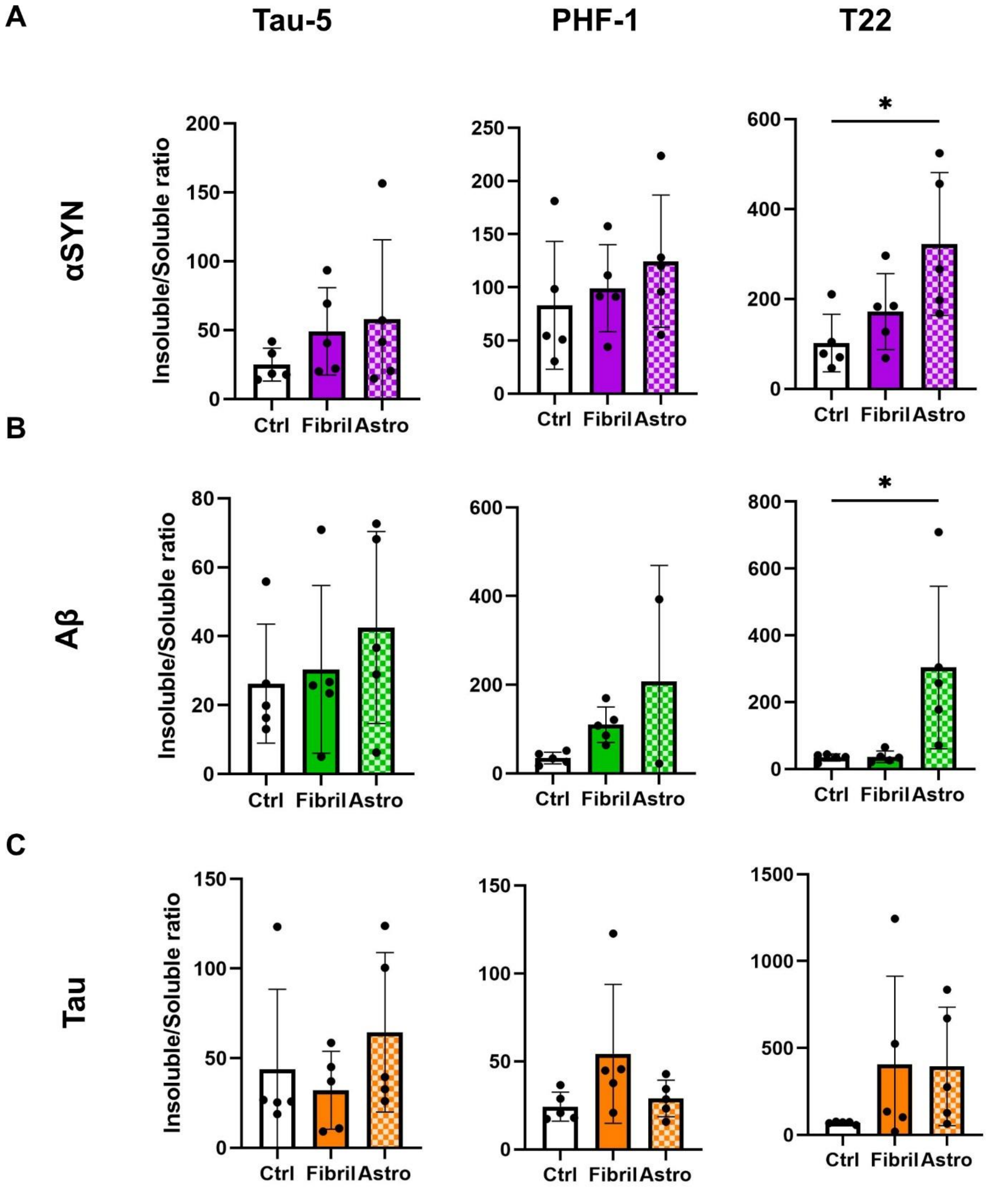

Figure S7. Ratio of the insoluble to soluble Tau pathology markers. Related to Figures 4 and 5. The ratio PHF-1, Tau-5 and T22 - positive signal in the insoluble fraction to that in the soluble fraction of each organoid lysate, as determined by western blot analysis. The panels correspond to (**A**) Quantification of αSYN blots. (**B**) Quantification of Aβ blots. (**C**) Quantification of Tau blots. n=5 individual organoids (Aβ-PHF-1 n=2 due to three samples having no detectable signal in the soluble fraction). Data is analysed by one-way ANOVA, with multiple comparisons relative to control and presented as mean +/- SD. P-values are presented as following; * $p < .05$, ** $p < .01$, *** $p < .005$, **** $p < .0001$.

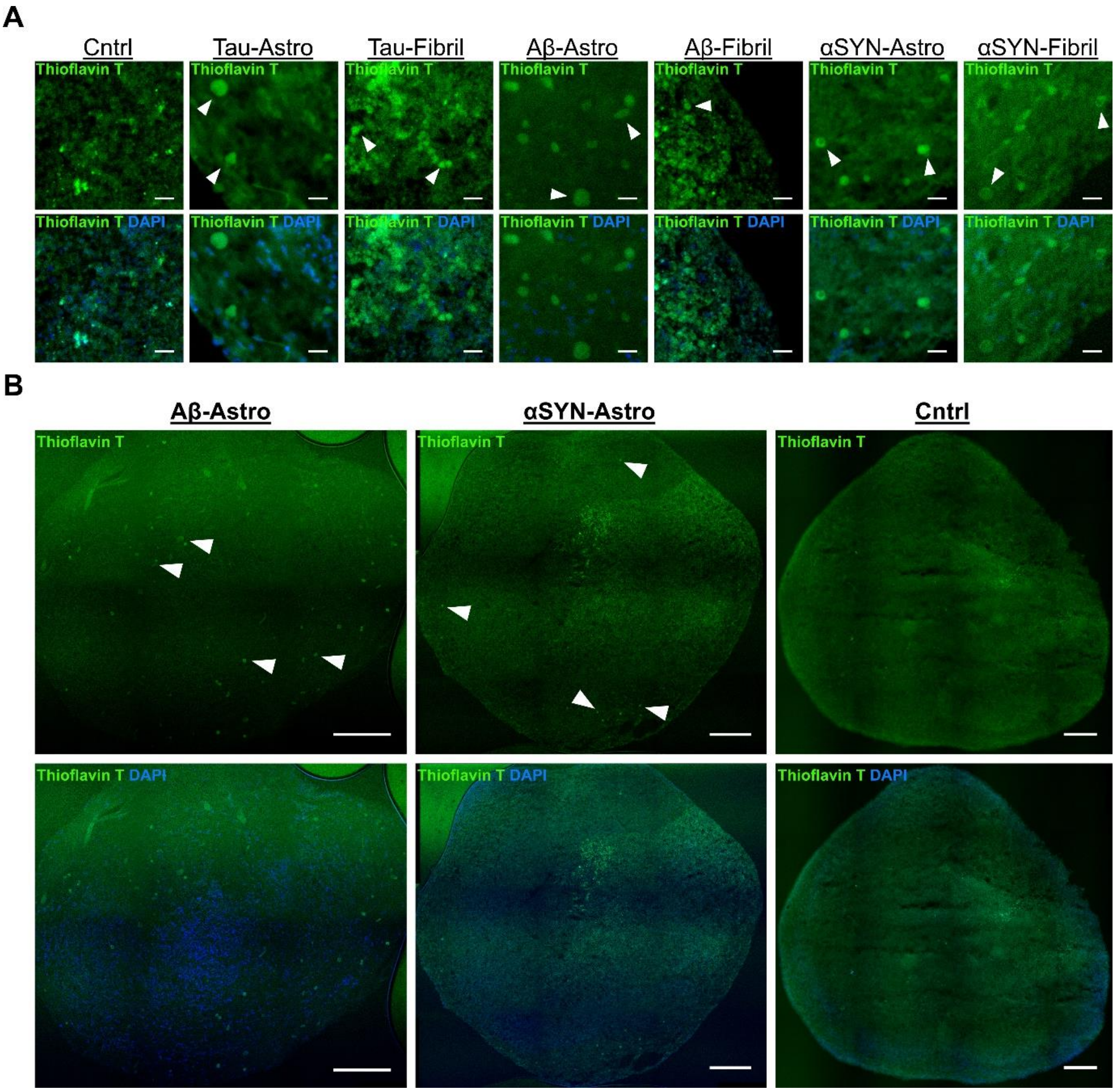

Figure S8. Fibril-exposed organoids contain Thioflavin T-positive structures. Related to Figures 4 and 5. (**A**) Thioflavin T staining of control organoids and organoids exposed to αSYN, Aβ, or tau fibrils, or to astrocytes pre-treated with the same proteins. The staining shows positive accumulations in the fibril-exposed organoids (arrows). (**B**) Distribution of Thioflavin T-positive aggregates (arrows) in the organoids exposed to astrocytes carrying Aβ or αSYN fibrils, but not in controls. Scale bars: 20 µm in A, 200 µm in B.

Figure 6A

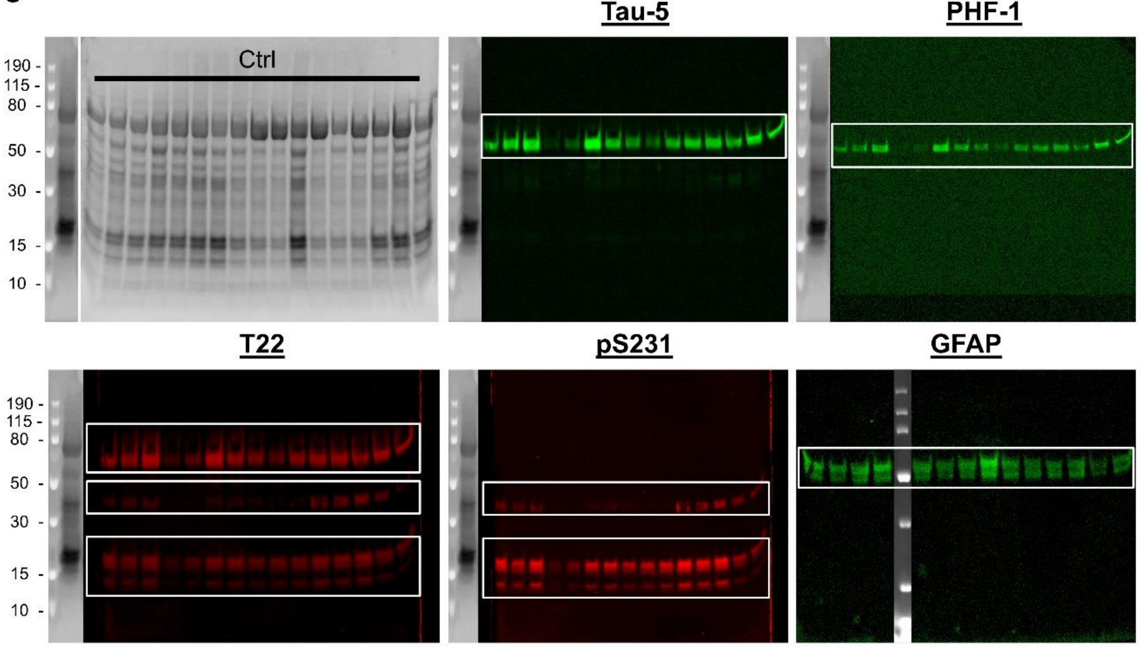

Figure 6E

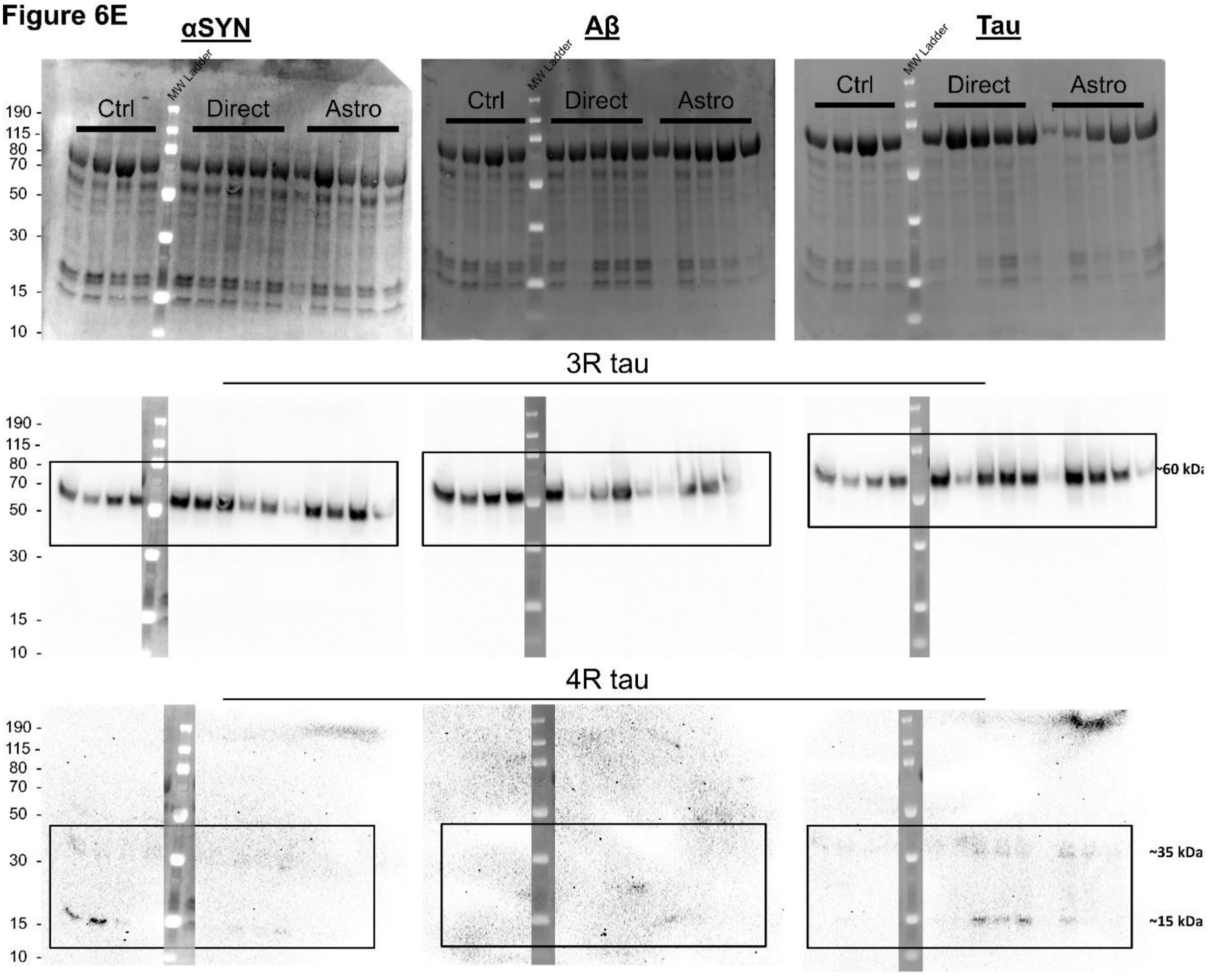

Figure S9. Related to Figure 6. Uncropped membranes and Nostain total protein loading controls of the membranes in Figure 6A-E. Figure 6A is a parallel of the control samples in Figure 4A and additional samples for analysis, so band sizes from the paired run were used as reference. For Figure 6E, the loading control (top panel) image was superimposed on each corresponding signal image (lower panels) to show the bands' location in reference to the MW ladder.
